# A Review of Judgments of Learning and Executive Functions in Spaced Learning: The Enemy of Spacing?

**DOI:** 10.7759/cureus.77230

**Published:** 2025-01-10

**Authors:** Xuechen Yuan

**Affiliations:** 1 Faculty of Education, University of Windsor, Windsor, CAN

**Keywords:** delayed jol, executive functions, jol reactivity, judgments of learning, spacing effect

## Abstract

The benefit of the spacing effect is inherently hindered by perception bias in making judgments of learning (JOLs), but more insights might be found in the context of executive functions (EF), where it correlates with metacognitive strategies and cognitive loads. Thus, this article attempts to address the dilemma of the spacing effect by synthesizing both existing JOL and EF perspectives. This paper yields various mechanisms related to memory performance in spaced learning: delayed JOLs, JOL reactivity, overt retrieval, inhibition control, working memory, and cognitive flexibility. All of these factors associate with the theory of mind, an important yet understudied social-cognitive skill in spaced learning which could shift our ways of thinking about spacing.

## Introduction and background

Learners develop competence and confidence during their study. Aside from getting good grades, learners need to believe they truly get what they have learnt. Currently, a common phenomenon in the classroom is cramming before exams, although it compromises long-term knowledge retention [1]. Numerous studies show that learners favour a cramming approach while undermining the benefits of spacing [2-5]. Regardless, spacing has been a promising learning practice that is resistant to forgetting, having been tested and replicated in at least 1000 experiments among different contexts and populations [6,7].

In a nutshell, spacing (as known as the spacing effect) works by dividing the learning sessions/materials in time or into short pieces, instead of presenting them at once or in a block order [8]. The benefits of spacing have been widely documented [6,9-12]. However, such a seemingly irrefutable learning strategy is fraught with complexities in memory monitoring biases, one of them being judgments of learning (JOLs), a predictive assessment made by learners during or after studying about the likelihood of their future memory retrieval [13,7]. This article primarily focuses on JOL, as it plays an essential role in memory retention [14,5,15]. Additionally, as a unique attempt, the paper would discuss how executive functions (EF) play a role in making JOL [16], given the scarcity of similar research [11].

## Review

Using the Google Scholar, PubMed, and PsycINFO databases, a detailed literature search was performed to identify original research studies directly addressing the role of JOL and EF in spaced learning. The thematic areas of this literature review were built upon the initial reading of existing publications, where the trends and gaps regarding cognitive mechanisms of spaced learning were identified. Next, for each section of the review, the search terms were applied differently. For related publications on applying JOL activities in spaced instruction, the search terms "JOL", "delayed JOL", "judgments of learning", "JOL reactivity", "spaced", "spacing", and/or "memory" were included to retrieve relevant studies. To understand how EF plays a role in JOL and memory biases, each of the three EF skills ("inhibition control", "working memory", and "cognitive flexibility") was searched with JOL by using AND operators. Articles on the relationship between EF and JOL were significantly lower in numbers. Overall, there were no restrictions on the types of curricula or study items used. Most papers were peer-reviewed articles published within 15 years alongside a few book chapters and dissertations. The final count of the literature analyzed was 64. A thorough summary and synthesis of findings from articles were conducted for this topic.

A review of JOLs in spaced instruction

The earliest study on JOL can be traced back to an unpublished experiment by Bower and Winchester in 1970 [17]. In the study, participants made JOLs (e.g., DOOR-?) after studying a series of pair associations (e.g., DOOR-RB), followed by a cue recall test after a five-minute break. The results from the experiment showed that their JOL ratings were far from consistent in predicting the actual recall test performance.

This early work inspired later JOL research. For instance, Kornell and Bjork conducted a study, asking participants to pair artworks with artists [3]. Although participants who received spaced learning (mixing artworks by different artists) performed better on memory tests, they rated massed learning (studying artworks by one artist consecutively) as more effective. Similar studies suggest that accessibility to short-term memory traces and temporary retrieval/processing fluency plays an important role in making JOLs [2,4]. The experience-based process of JOL supports this claim [7]. That is, to avoid quick forgetting of weakly encoded items, participants subconsciously use massing to minimize the perceptual distance of these items with their initial presentations. This is especially true for difficult or high-priority items, requiring more retrieval efforts, which creates a momentary "illusion of confidence" that they can remember these items in the future [2,18]. Learners' past successes with massing reinforce their misinformed beliefs on how memory works and their perceived memory (in)competence [7]. Thus, the massing bias hinders the predictive accuracy of massed JOL in relation to their actual memory performance.

Early studies by Nelson and Dunlosky [17] and Dunlosky and Nelson [19,20] argue that the predictive accuracy of JOL relies on specific conditions. For example, these findings showed greater consistency between JOLs and subsequent memory measures when JOLs were created at a delay (with a retention interval) following the initial study and when only the cue was displayed during JOL (i.e., cue-only; ART-?) compared to a cue-target JOL (ART-girl) made immediately after studying. This strong association is dubbed as the delayed-JOL effect or sometimes the delayed cue-only JOL effect. So how does the delayed-JOL effect inform the spacing effect? Two issues were at hand: (1) delayed JOLs predict memory retention and (2) delayed JOLs affect memory retention.

Delayed JOLs Predict Memory Retention: Spaced Retrieval

Delayed JOL (spaced) allows learners to predict the future success of memory retrieval more accurately compared to immediate or massed JOLs collected by learners after the initial presentation of study items [21]. This is because delayed JOLs are made from accessibility to already consolidated memory, while immediate JOLs are based on biased perception fluency of transient memory cues, which may fade before the test [17]. For example, the underconfidence-with-practice effect suggests that learners may overestimate their memory retention during initial immediate JOL, but each rehearsal of the same study items (spaced) reduces the confidence of memory prediction when learners slowly become aware that their JOLs lag behind their actual memory test performance [22].

To illustrate the effect of perception biases on JOL and how delayed JOL attenuates it, a meta-analysis by Luna et al. reviewed 28 experiments that present study items in either small or large font size (to imitate a sense of priority), followed by making delayed or immediate JOLs, restudy decisions, and finally a memory test [21]. They found that a delay slightly reduced the font size effect on memory, but greatly diminished its effect on JOLs and restudy decisions. That is, while participants remembered large font words better after a delay, they did not attribute their better memory to the perceptual advantage of large font sizes. As the perceptual characteristics of items fade rapidly, retrieval practices tend to rely more on accessibility to long-term memory, making delayed JOL a better predictor of actual memory retention [21].

Despite the advantage, the predictive accuracy of delayed JOL depends on task demand and level of retrieval. Regarding task demand, assuming that the memory test relies on retrieval from long-term memory, immediate JOL may be as accurate as delayed JOL should study items be consolidated into long-term memory at the time of JOL [23,17]. Bui et al. examine this by adding secondary tasks (e.g., math problems) to fill the retention interval between studying and JOL [23]. The idea is to know if the JOL accuracy increases when secondary tasks displace study items from short- to long-term memory (i.e., displaced-JOL effect). The results indicate that immediate and delayed JOL show comparable accuracy only when secondary tasks are sufficiently demanding, supporting the notion that task demands correlate with JOL accuracy via reliance on long-term memory retrieval.

It is also worth noting that both immediate and delayed JOLs may not demonstrate the same level of retrieval effort as would be expected on an actual memory test [23]. At the bottom line, JOL tends to evoke covert retrieval, with immediate JOL assessed predominantly from short-term memory and delayed JOL from long-term memory via spaced retrieval. On the other hand, testing elicits overt retrieval. A few studies have illustrated the testing effect in pair-associate learning [24-26,15]. For example, Jönsson et al. [24] and Putnam and Roediger [25] found that both delayed JOL (covert) and overt retrieval (e.g., recall target words given the cues) yield comparable recall efficiency on cue-recall tests. However, using a single study-test learning trial, Tauber et al. found that overt retrieval outperforms delayed and immediate JOLs and more time was spent on overt retrieval practices [26]. The results imply that learners do not engage in full retrieval during delayed JOLs. Incorporating restudy as a control, Tekin and Roediger found that cue-only delayed JOL predicts recall better than restudy, but worse than overt rehearsal, because participants in delayed JOL tended to truncate retrieval for unfamiliar cues [15]. Overall, these previous studies highlight the importance of the testing effect on memory retention.

Alongside the spaced retrieval mechanism of delayed JOLs, Tekin and Roediger also shed light on the potential confounding role of the non-retrieval (strategic) mechanism in the cue-target JOL condition [15]. That is, showing both cue and target simultaneously may help participants form more transient behavioural comfort with related study items, rather than engaging in a deep long-term memory search. In the study, the confounding effect of the non-retrieval mechanism was insignificant as cue-target delayed JOL did not outperform the restudy condition on recall. However, this finding prompts this paper to segue into how making delayed JOLs not only predicts but also affects memory test performance.

Delayed JOLs Affect Memory Retention: JOL Reactivity

The act of making JOL intentionally or unintentionally affects memory retention, a phenomenon termed JOL reactivity, which has been relevant since the delayed JOL effect was initially discovered [27,15]. To elucidate this, a meta-analysis by Double et al. highlighted that making immediate JOLs leads to a moderate positive JOL reactive effect on cue-recall performance (i.e., JOL increases memory retention), especially for strongly semantic-related cue-target pairs [28]. Furthermore, Soderstrom et al. found that immediate JOLs benefit memory recall compared to not making JOLs [29]. However, Chilcott's systematic review of 44 experiments demonstrated that the positive reactive effect of immediate JOL is not as strong as delayed JOL [30].

The role of word-pair relatedness is evident in broader JOL reactivity literature, where JOL facilitates the retention of highly related word-pair items [30]. For example, Myers et al. found that JOL reactivity is strongest in cue-recall tests, compared to any other tasks like recognition and free recall [31]. A lack of reactivity, sometimes even negative reactivity (i.e., JOL reduces memory retention) for unrelated items, is also found in studies by Janes et al. [32] and Maxwell and Huff [33]. The dual-task hypothesis underlying the findings suggests that making JOLs produces additional cognitive demands for memory retention that hinder cue recall for difficult-to-be-retrieved items [27]. Regardless, the phenomenon of JOL reactivity is complex with inconsistent findings. Opposite to Chilcott's systematic review [30], Witherby and Tauber found that making JOL benefits the long-term retention of related paired items regardless of whether JOL was delayed or immediate, implying that the mechanism of relational encoding via JOL appears for both immediate and delayed conditions [34].

The discrepancy in results may stem from methodological differences [32]. Compared to the majority of studies using experimenter-paced learning procedures, Mitchum et al. showed that different methods such as self-paced learning may elicit alternative mechanisms underlying JOL reactivity [35]. During self-regulated learning with personalized study time, making JOL shifts one's subsequent study strategies, prompting them to either prioritize more related/easier word pairs [32,35] or reduce efforts on learning related items that were "too easy", surprisingly leading to negative reactivity [36].

Nevertheless, further research by Rivers et al. [37] challenges the notion that making JOL shifts learners' study strategies, by finding no significant difference in self-reported learning strategies between JOL and control conditions. They argue that if strategy shifts indeed drive memory performance, then all related word pairs following the shifts (even without making JOL) shall benefit from the updated strategies. Currently, there is a lack of consistent evidence on whether JOL reactivity results from non-retrieval benefits akin to learning habit shifts, requiring further research.

The role of EF on JOL

EF are core abilities for learning and scholastic performance, referring to a set of metacognitive processes beyond instinct and intuition, which help people maintain focus and control and make more informed decisions. These processes include inhibition control, working memory, and cognitive flexibility, which have been defined more broadly that appear synonymous with each other [16]. A link between EF and metamemory judgment has been previously identified by Souchay et al. [38].

Inhibition Control

Inhibition control, or suppression of internal predispositions or distractor stimuli, enables people to maintain focused attention to align their actions with voluntary and endogenous goals [39]. Previous studies by Metcalfe and Xu [40] and Xu and Metcalfe [41] suggest that learners stay focused and engaged when instruction schedules and task difficulties are calibrated to their current region of proximal learning, aligning with previous learning theories by Piaget [42] and Vygotsky [43]. For example, they found that mind-wandering (ceasing behavioural control) frequently occurs in massed/blocked instructions and learning items inconsistent with individuals' levels of mastery. Only difficult items that are of high value are allocated more attention that partially alleviates mind-wandering [44].

Regarding inhibition in the level of cognition, a more recent study by Park et al. [11] examines the role of EF in influencing the efficiency of different instruction strategies in science learning among middle school students. They found that EF is associated with memory recall, driven by controlled retrieval, and not associated with the recognition test which relies more on cue familiarity [45]. Specifically, inhibition control correlates with better memory recall in interleaved (spaced) instruction, where different topics are intermixed. Interleaved practices allow learners to practice more active retrieval and shift attention across topics, rather than relying on passive learning [11]. Unlike in massed instruction, learners in spaced sessions are required to intentionally resist unwanted and prepotent memories such as recently learnt items (proactive interference) and retroactive interference from soon-to-be learnt items [16,46].

Numerous articles suggest that learners may underestimate (make poor JOL of) spaced practice due to its costly strategic and attentional demands [3,40,47]. Carvalho and Goldstone demonstrate that attentional bias affects significantly how learners judge the benefits of study schedules when learners are adapted to unsupervised learning that promotes automatic and passive rule-based categorizations of study items, which encourages learners to focus on similarities rather than update attention to distinguish between items [48]. On a positive note, Was et al. found that high JOLs are associated with less mind-wandering, indicating that learners are aware of the negative effects of ceasing attention [49]. They also make higher JOLs for mind-wandering activities that are more closely related to tasks, compared to complete task-unrelated thoughts.

It is also important to note the role of attention in JOL reactivity. While making JOLs helps sustain attention and process information more thoroughly [50,51], such benefits are not replicated in all experiments such as Dougherty et al. [52] and Rivers et al. [27] that employ attention-reorienting tasks to regulate the amount of attention used in making JOLs. Simply put, they found that JOLs improve memory recall, but this benefit is not strongly accounted for by enhanced attention demands during encoding and making JOLs. Word-pair relatedness (or cue-strengthening hypothesis) remains the dominant explanation for JOL reactivity in literature [29]. It implies that a more controlled, mindful, and inductive learning approach might facilitate unlearning and intentional forgetting more than it does for rote memorization and strengthening cues, strategically speaking, might still be beneficial for long-term retention.

Working Memory

Distinct from short-term memory, working memory involves not only "holding information in mind" but also "mentally working with it…[and] relating that to what comes later" [16]. It is a core EF for it facilitates planning, problem-solving, and decision-making by mentally reasoning, operating, updating, and integrating. Working memory often co-exists with inhibition control, responsible for holding information to guide inhibition and using inhibition to discipline working memory [53]. Currently, the role of working memory in differential study schedules is unclear, with a lack of consistent evidence predicting its effect [11,54,55]. One explanation for the role of working memory is that it predicts learning in blocked but not interleaved practices because higher working memory demand in interleaved instruction (updating working memory of mixed topics and integrating them) may deplete learners' cognitive resources [54,56]. This is consistent with studies that find negative correlations between cognitive load and JOLs [57].

Not surprisingly, existing studies underline the tension between JOL and working memory. For example, Yang et al. argue that items with high JOLs are likely easier to process, which spares the working memory-driven metacognitive control network [58]. However, when working memory demand increases in learning ambiguous materials and performing complex tasks, making JOLs without deep processing can become overwhelming and thus impede learning, unless accompanied by overt retrieval practices [59]. For high working memory-loaded tasks such as arithmetic problem-solving, Baars et al. argue that delayed JOLs (judging comprehension of procedures) may not predict the quality of mathematics learning as accurately as for pair-associate learning [57]. Immediate JOLs, on the contrary, appear to better reflect the complexity of mathematical reasoning as the salience of mental representations (procedure schema) decays rapidly. When knowledge is formed with elements updated and integrated into a procedural schema, working memory demands and cognitive load decrease, allowing learners to make higher and more accurate JOLs [60]. These findings are consistent with Rohrer et al. who argue that a small block of mathematics problems created immediately after the study might optimize learning, as it allows learners to familiarize themselves with the procedural steps before memory decays, though massed repetition can still impede learning [61].

Cognitive Flexibility

Cognitive flexibility is a difficult concept to describe due to a lack of a unified definition and many factors to be considered [16]. Regardless, the skill of shifting (switching perspectives and task-switching) is often used to represent cognitive flexibility. Shifting is a valid predictor of self-regulated learning, scientific reasoning skills, and memory performance from interleaved practices [62,11].

In everyday life, people often partake in activities that require multitasking (parallel processing) and task-switching (serial processing). Doing so, multitaskers, particularly self-initiated, are often unaware of its negative effects and wrongly believe that they perform well (i.e., high JOLs) in shifting between tasks, despite research showing that multitasking is associated with more mind-wandering and lower task-switching accuracy [63,64]. Multitaskers tend to make higher JOLs when they are able to stay on-task compared to losing control of their thoughts [49]. Overall, learners' judgment of shifting appears to be related to how shifting and distractions initiate.

There is a scarcity of research directly investigating the relationship between shifting and JOL. One popular explanation for this gap is that JOL, even with delayed, depends more on cue relatedness and ease of recall, a memory-association mechanism that is less relevant to cognitive flexibility that focuses on executive operation [38]. Regardless, similar studies have examined attributes closely related to JOL, such as feeling of knowing (FOK): the judgment made at the retention phase on the likelihood one would later recognize an item they currently fail to retrieve [65,66]. FOK involves a certain degree of long-term memory retrieval into working memory, which also shares a common neural process with task-switching: retrieving task sets to operate them in working memory [67].

Among EF, shifting is the core component that leads to accurate FOK about whether an item is stored in episodic memory [66]. This is because shifting aids in memory search and holding partially retrieved information in working memory while switching mental representations back and forth to judge the possibility of using this information for future recognition [68]. Building on it, Boduroglu et al. emphasize the need to dissect shifting function into switching and multitasking, instead of treating it as a unified composite [65]. For example, they found that FOK accuracy was associated with the cognitive demands of performing task-switching but not multitasking. But more surprisingly, they found that demands of task-switching do not predict actual memory performance, implying that shifting and other EF may not play important roles in memory retrieval [65].

Intersecting themes: theory of mind (ToM)

Across diverse contexts, EF such as inhibition control, working memory, and cognitive flexibility demands a high level of mindfulness and perspective-taking, similar to ToM that addresses the general ability of individuals to attribute cognitive and affective mental states to themselves and others [69,70]. Development of EF and ToM ability often intersects throughout preschool years and adulthood due to the shared neural processing in the prefrontal cortex [69]. Future studies on ToM might have great potential for understanding learners' metacognitive attribution of learning.

Lockl and Schneider identified a tangible link between ToM and JOL performance in neurotypical developing children, both involving metacognitive control [71]. In particular, early language and ToM development account for significant variance in one's future self-assessment of memory processes. Similarly, Wang and Fyre identified an association between children's learning comprehension (e.g., intention understanding and knowledge change) and ToM development, a mechanism of monitoring mental states during learning which extends beyond memory recall prediction like JOL [72].

Vygotsky believes that mediated and supervised learning fosters a "non-spontaneous" mode of information processing [73]. However, when learners are burdened with learning demands, reverting to unsupervised learning that promotes priming, automation, and rule-based instruction becomes a tempting choice [48,73]. Learning is beyond mere rote memorization of study items; thus, it is important to integrate ToM activities through mediated and supervised learning to allow learners to create their mentalistic explanation of learning, recognize different orders of false beliefs in learning such as massing bias and overconfidence, as well as construct useful strategies for life-long (self-regulated) learning to overcome these false beliefs.

## Conclusions

This article discusses two important factors of spaced learning: JOL and EF. Recent JOL perspectives indicate that the predictive accuracy of JOL on memory performance relies on its timing, task demands, and depths of memory retrieval. Besides predicting, JOL activities also actively affect learning via cue-strengthening hypothesis and strategic shifts in learning, although these perspectives remain debated. EF plays a crucial role in JOL accuracy. Spaced learning often requires higher strategic, attention, and working memory demands, which are central EF components. Recent EF perspectives highlight the non-memory-strengthening role of EF in spaced learning and its plausible EF cognitive costs while spacing, implying that the relationship between EF and memory is not linear. Mindful and controlled monitoring of learning is a product of optimal EF demands. While needed to be treated with caution, EF demands partially explain how massing bias is formed. Massing bias also arises from over-emphasis on remembering what is learnt. Finally, integrating ToM perspectives into teaching with a de-focus on memorization might alleviate the EF demands, optimizing the learning context where the spacing effect could become more useful.
